# 5-(Prop-2-yn-1-yl)-5*H*-dibenzo[*b*,*f*]azepine: ortho­rhom­bic polymorph

**DOI:** 10.1107/S1600536812048908

**Published:** 2012-12-05

**Authors:** M. M. M. Abdoh, S. Madan Kumar, K. S. Vinay Kumar, B. C. Manjunath, M. P. Sadashiva, N. K. Lokanath

**Affiliations:** a Department of Physics, Faculty of Science, An Najah National University, Nabtus West Bank, Palestine; b Department of Studies in Physics, Manasagangotri, University of Mysore, Mysore 570 006, India; c Department of Studies in Chemistry, Manasagangotri, University of Mysore, Mysore 570 006, India

## Abstract

In the title ortho­rhom­bic polymorph (space group *Iba*2), C_17_H_13_N, the dihedral angle between the benzene rings is 55.99 (10)° and the azepine ring adopts a boat conformation. In the crystal, mol­ecules are linked by C—H⋯π contacts. The previously-reported polymorph [Yousuf *et al.* (2012 ▶). *Acta Cryst*. E**68**, o1101] crystallizes in the monoclinic system (space group *P*2_1_/*c*) with two mol­ecules in the asymmetric unit.

## Related literature

For the previously-reported monoclinic polymorph, see: Yousuf *et al.* (2012 ▶). For biochemical background, see: Sadashiva *et al.* (2005 ▶).
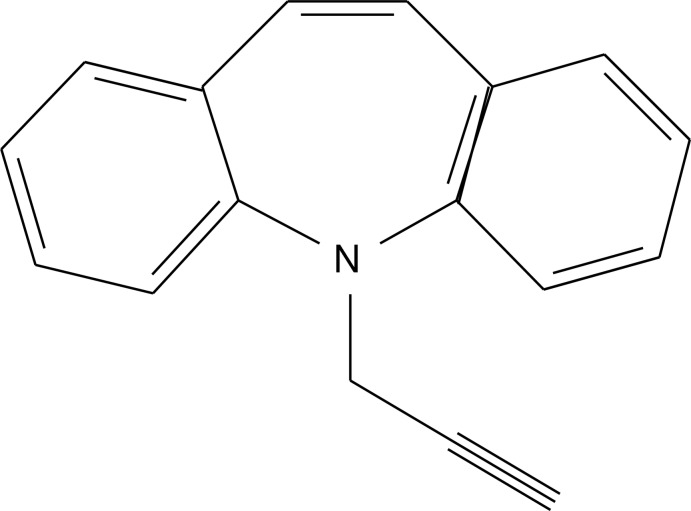


## Experimental

### 

#### Crystal data

C_17_H_13_N
*M* *_r_* = 231.28
Orthorhombic, 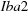

*a* = 16.2444 (6) Å
*b* = 21.1700 (6) Å
*c* = 7.2399 (2) Å
*V* = 2489.76 (13) Å^3^
*Z* = 8
Mo *K*α radiation
μ = 0.07 mm^−1^
*T* = 103 K
0.35 × 0.30 × 0.25 mm

#### Data collection

Oxford Diffraction Xcalibur Eos diffractometer
10081 measured reflections
1199 independent reflections
1132 reflections with *I* > 2σ(*I*)
*R*_int_ = 0.027

#### Refinement

*R*[*F* ^2^ > 2σ(*F* ^2^)] = 0.026
*wR*(*F* ^2^) = 0.067
*S* = 1.09
1199 reflections
164 parameters
1 restraint
H-atom parameters constrained
Δρ_max_ = 0.08 e Å^−3^
Δρ_min_ = −0.10 e Å^−3^

### 

Data collection: *CrysAlis PRO* (Oxford Diffraction, 2009 ▶); cell refinement: *CrysAlis PRO*; data reduction: *CrysAlis PRO*; program(s) used to solve structure: *SHELXS97* (Sheldrick, 2008 ▶); program(s) used to refine structure: *SHELXL97* (Sheldrick, 2008 ▶); molecular graphics: Mercury (Macrae *et al.*, 2006 ▶); software used to prepare material for publication: *SHELXL97*.

## Supplementary Material

Crystal structure: contains datablock(s) global, I. DOI: 10.1107/S1600536812048908/hb6998sup1.cif

Structure factors: contains datablock(s) I. DOI: 10.1107/S1600536812048908/hb6998Isup2.hkl

Supplementary material file. DOI: 10.1107/S1600536812048908/hb6998Isup3.cml

Additional supplementary materials:  crystallographic information; 3D view; checkCIF report

## Figures and Tables

**Table 1:** Hydrogen-bond geometry (Å, °) *Cg*1 and *Cg*2 are the centroids of the C2/C3/C12–C15 and C6-C11 rings, respectively.

*D*—H⋯*A*	*D*—H	H⋯*A*	*D*⋯*A*	*D*—H⋯*A*
C10—H10⋯*Cg*2^i^	0.95	2.75	3.659 (2)	160
C18—H18⋯*Cg*1^ii^	0.95	2.58	3.512 (2)	167

Symmetry codes: (i) 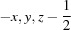; (ii) 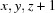.

**Fig. 1.**
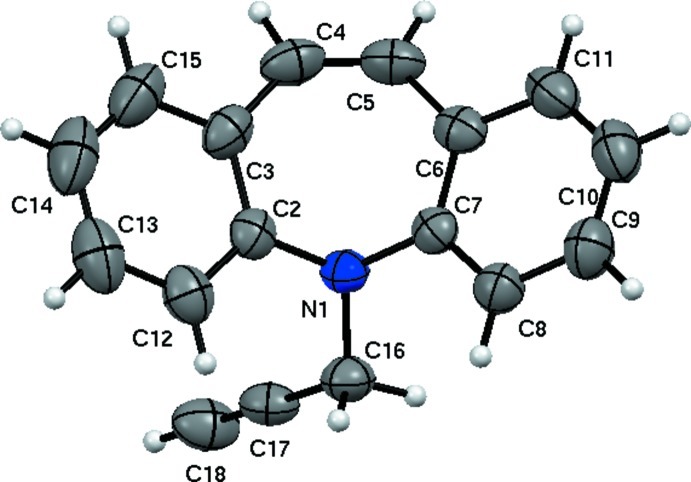
The molecular structure of the title molecule with 50% probability ellipsoids.

**Fig. 2.**
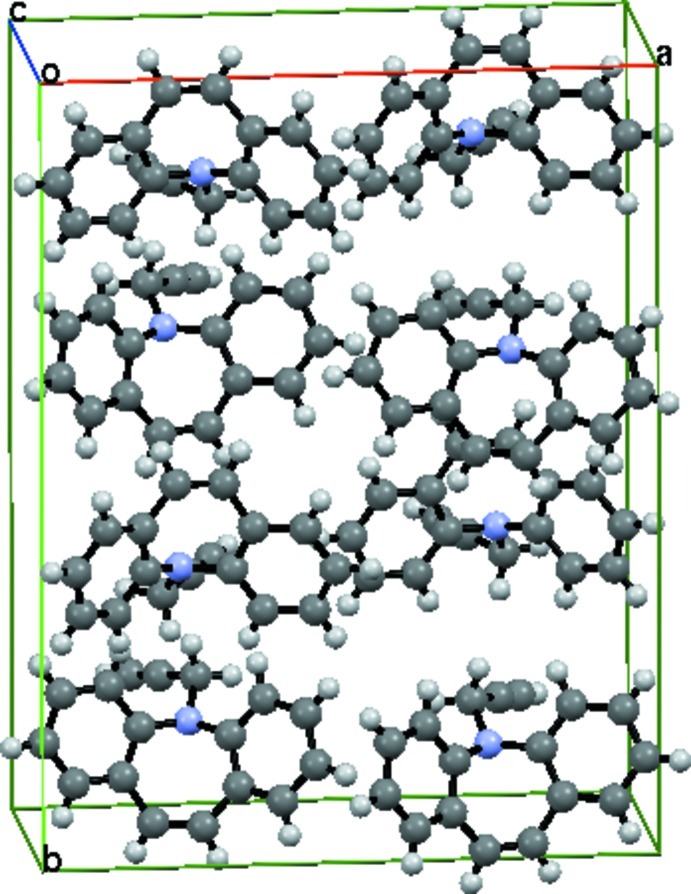
Packing diagram of molecule, viewed along the crystallographic a axis. Dashed lines indicates short contacts.

## supplementary crystallographic information

### Comment

As part of our studies of new derivatives useful for tailoring of biologically active 5-membered heterocyclic rings such as 3,5-disubstituted isoxazole (Sadashiva *et al.*, 2005), we now describe the title compound.

In the title molecule, C_17_ H_13_ N (Fig. 1.), benzene rings fused to azepine rings are nearly planar and its geometry is similar to 5-(Prop-2-ynyl)-5*H*-dibenzo[*b,f*]azepine (Yousuf *et al.*, 2012). The dihedral angle between the benzene rings is 55.99 (10)° which is almost equal and large compared to the two molecules repectively for the first polymorph.

Seven-membered azepine ring adopts a boat conformation as indicated by the puckering parameters Q_2_ = 0.7126 (18) Å, Q_3_ = 0.2154 (17) Å, φ_2_ = 177.93 (15) °, φ_3_ = 178.1 (5) °, and the total puckering amplitude Q_T_ = 0.7444 (17) Å. The title molecule adopts butterfly shape which may be essential for inhibition pocket which is similar to the reported polymorph (Yousuf *et al.*, 2012).

The packing of the title molecules is as shown (Fig. 2.) and features short C—H···*π* contacts (Table 1).

### Experimental

5*H*-dibenzo[b,f]azepine (1, 0.0026 mol) was taken in a mixture of toluene and water in the ratio 1:1,was added sodium hydroxide (0.026 mol) followed by tetra-n-butylammonium bromide (TBAB) (0.00286 mol) at room temperature. After 15 minutes, propargyl bromide was added (0.00286 mol) at room temperature. Then, the resulting reaction mixture was heated at 60°C for 5 h. After completion of reaction (monitored by TLC), the reaction mixture was diluted with water (50 ml). The aqueous layer was extracted with ethyl acetate (3 × 20 ml), the combined ethyl acetate layer was washed with 0.1 N hydrochloric acid (2 × 25 ml), followed by brine solution (2 × 25 ml). Then, the organic layer was dried over anhydrous sodium sulfate, filtered and concentrated under reduced pressure to afford crude 2, which was purified by column chromatography over silica gel (60–120 mesh) using Hexane: Ethyl acetate mixture in 9.5:0.5 ratios as eluent. The pure compound 2 was crystallized in ethyl acetate and hexane to obtain yellow blocks.

^1^H NMR (DMSO-d6, 300 MHz): δ 7.33 (t, J=7.2 Hz, 2H), 7.13 (t, J=8.7 Hz, 4H), 7.02 (t, J=7.2 Hz, 2H), 6.75 (s, 2H), 4.51 (d, J=1.8 Hz, 2H), 3.08 (s, 1H).

MS (*M*^+^+1): 232. Melting point (°C): 90 (Uncorrected)

### Refinement

In the absence of significant anomalous dispersion effects Friedel pairs were merged. All the hydrogen atoms of the compound are fixed geometrically (C—H= 0.93–0.97 Å) and allowed to ride on their parent atoms. The poorly fitted reflections (2 0 0) and (1 1 0) were omitted during refinement.

### Crystal data

**Table:**

C_17_H_13_N	*F*(000) = 976
*M**_r_* = 231.28	*D*_x_ = 1.234 Mg m^−^^3^
Orthorhombic, *I**b**a*2	Mo *K*α radiation, λ = 0.71073 Å
Hall symbol: I 2 -2c	Cell parameters from 1199 reflections
*a* = 16.2444 (6) Å	θ = 3.2–25.0°
*b* = 21.1700 (6) Å	µ = 0.07 mm^−^^1^
*c* = 7.2399 (2) Å	*T* = 103 K
*V* = 2489.76 (13) Å^3^	Block, yellow
*Z* = 8	0.35 × 0.30 × 0.25 mm

### Data collection

**Table:**

Oxford Diffraction Xcalibur Eos diffractometer	1132 reflections with *I* > 2σ(*I*)
Radiation source: fine-focus sealed tube	*R*_int_ = 0.027
Graphite monochromator	θ_max_ = 25.0°, θ_min_ = 3.2°
Detector resolution: 16.0839 pixels mm^-1^	*h* = −19→19
ω scans	*k* = −25→25
10081 measured reflections	*l* = −8→8
1199 independent reflections	

### Refinement

**Table:**

Refinement on *F*^2^	Secondary atom site location: difference Fourier map
Least-squares matrix: full	Hydrogen site location: inferred from neighbouring sites
*R*[*F*^2^ > 2σ(*F*^2^)] = 0.026	H-atom parameters constrained
*wR*(*F*^2^) = 0.067	*w* = 1/[σ^2^(*F*_o_^2^) + (0.0354*P*)^2^ + 0.3725*P*] where *P* = (*F*_o_^2^ + 2*F*_c_^2^)/3
*S* = 1.09	(Δ/σ)_max_ < 0.001
1199 reflections	Δρ_max_ = 0.08 e Å^−^^3^
164 parameters	Δρ_min_ = −0.10 e Å^−^^3^
1 restraint	Extinction correction: *SHELXL97* (Sheldrick, 2008), FC^*^=KFC[1+0.001XFC^2^Λ^3^/SIN(2Θ)]^-1/4^
Primary atom site location: structure-invariant direct methods	Extinction coefficient: 0.0046 (6)

### Special details

**Table:**

Geometry. Bond distances, angles *etc*. have been calculated using the rounded fractional coordinates. All su's are estimated from the variances of the (full) variance-covariance matrix. The cell e.s.d.'s are taken into account in the estimation of distances, angles and torsion angles
Refinement. Refinement on *F*^2^ for ALL reflections except those flagged by the user for potential systematic errors. Weighted *R*-factors *wR* and all goodnesses of fit *S* are based on *F*^2^, conventional *R*-factors *R* are based on *F*, with *F* set to zero for negative *F*^2^. The observed criterion of *F*^2^ > σ(*F*^2^) is used only for calculating -*R*-factor-obs *etc*. and is not relevant to the choice of reflections for refinement. *R*-factors based on *F*^2^ are statistically about twice as large as those based on *F*, and *R*-factors based on ALL data will be even larger.

### Fractional atomic coordinates and isotropic or equivalent isotropic displacement parameters (Å^2^)

**Table:**

	*x*	*y*	*z*	*U*_iso_*/*U*_eq_	
N1	0.23053 (8)	0.36797 (6)	0.6618 (2)	0.0409 (4)	
C2	0.31311 (10)	0.36894 (7)	0.5957 (3)	0.0414 (5)	
C3	0.34432 (10)	0.42627 (8)	0.5292 (3)	0.0492 (6)	
C4	0.29519 (12)	0.48379 (8)	0.5170 (3)	0.0560 (6)	
C5	0.21579 (11)	0.48775 (7)	0.4806 (3)	0.0538 (6)	
C6	0.15967 (10)	0.43569 (8)	0.4423 (3)	0.0450 (5)	
C7	0.16937 (9)	0.37583 (7)	0.5219 (2)	0.0399 (5)	
C8	0.11790 (10)	0.32691 (8)	0.4686 (3)	0.0483 (6)	
C9	0.05556 (11)	0.33719 (9)	0.3419 (3)	0.0592 (7)	
C10	0.04355 (11)	0.39633 (10)	0.2688 (3)	0.0635 (7)	
C11	0.09515 (11)	0.44467 (9)	0.3192 (3)	0.0571 (6)	
C12	0.36307 (11)	0.31589 (9)	0.5992 (3)	0.0532 (6)	
C13	0.44347 (12)	0.31917 (11)	0.5374 (3)	0.0705 (8)	
C14	0.47460 (12)	0.37508 (13)	0.4711 (4)	0.0791 (9)	
C15	0.42559 (12)	0.42769 (11)	0.4679 (4)	0.0691 (8)	
C16	0.21109 (11)	0.32276 (8)	0.8082 (3)	0.0491 (6)	
C17	0.26199 (11)	0.33371 (8)	0.9703 (3)	0.0474 (6)	
C18	0.30303 (14)	0.34250 (9)	1.1020 (3)	0.0616 (7)	
H4	0.32340	0.52250	0.53740	0.0670*	
H5	0.19270	0.52900	0.47920	0.0650*	
H8	0.12550	0.28600	0.51950	0.0580*	
H9	0.02100	0.30320	0.30540	0.0710*	
H10	−0.00010	0.40370	0.18410	0.0760*	
H11	0.08650	0.48550	0.26830	0.0690*	
H12	0.34190	0.27700	0.64430	0.0640*	
H13	0.47730	0.28260	0.54070	0.0850*	
H14	0.52970	0.37720	0.42790	0.0950*	
H15	0.44760	0.46630	0.42260	0.0830*	
H16A	0.15230	0.32680	0.84240	0.0590*	
H16B	0.22030	0.27930	0.76240	0.0590*	
H18	0.33610	0.34960	1.20820	0.0740*	

### Atomic displacement parameters (Å^2^)

**Table:**

	*U*^11^	*U*^22^	*U*^33^	*U*^12^	*U*^13^	*U*^23^
N1	0.0432 (7)	0.0429 (7)	0.0367 (8)	−0.0037 (6)	0.0005 (6)	0.0061 (6)
C2	0.0424 (8)	0.0505 (9)	0.0314 (8)	−0.0034 (8)	−0.0036 (8)	−0.0045 (7)
C3	0.0494 (9)	0.0594 (10)	0.0389 (10)	−0.0153 (8)	−0.0019 (8)	−0.0043 (8)
C4	0.0762 (12)	0.0439 (10)	0.0479 (11)	−0.0187 (8)	0.0020 (10)	−0.0015 (8)
C5	0.0747 (11)	0.0393 (9)	0.0474 (11)	0.0003 (8)	0.0066 (11)	0.0039 (8)
C6	0.0506 (9)	0.0464 (8)	0.0380 (9)	0.0061 (7)	0.0079 (8)	0.0023 (8)
C7	0.0385 (8)	0.0449 (8)	0.0362 (9)	0.0031 (7)	0.0050 (7)	−0.0001 (7)
C8	0.0464 (9)	0.0486 (9)	0.0498 (11)	−0.0009 (8)	−0.0006 (9)	−0.0001 (8)
C9	0.0498 (10)	0.0693 (12)	0.0584 (13)	−0.0044 (9)	−0.0072 (10)	−0.0051 (10)
C10	0.0506 (10)	0.0866 (14)	0.0533 (13)	0.0091 (11)	−0.0072 (10)	0.0044 (12)
C11	0.0574 (11)	0.0628 (11)	0.0511 (11)	0.0143 (9)	0.0018 (10)	0.0122 (9)
C12	0.0539 (10)	0.0610 (11)	0.0448 (11)	0.0056 (9)	−0.0074 (9)	−0.0068 (9)
C13	0.0560 (11)	0.1000 (17)	0.0554 (13)	0.0212 (12)	−0.0066 (11)	−0.0157 (13)
C14	0.0453 (11)	0.131 (2)	0.0610 (14)	−0.0082 (12)	0.0035 (11)	−0.0112 (15)
C15	0.0540 (11)	0.0919 (15)	0.0615 (13)	−0.0257 (11)	0.0031 (11)	−0.0036 (13)
C16	0.0545 (10)	0.0483 (9)	0.0446 (11)	−0.0065 (8)	−0.0006 (9)	0.0095 (8)
C17	0.0632 (11)	0.0407 (8)	0.0384 (10)	−0.0004 (8)	0.0064 (10)	0.0056 (8)
C18	0.0885 (15)	0.0558 (11)	0.0404 (11)	−0.0061 (11)	−0.0077 (11)	0.0014 (9)

### Geometric parameters (Å, º)

**Table:**

N1—C2	1.424 (2)	C14—C15	1.369 (3)
N1—C7	1.429 (2)	C16—C17	1.454 (3)
N1—C16	1.463 (2)	C17—C18	1.178 (3)
C2—C3	1.401 (2)	C4—H4	0.9500
C2—C12	1.386 (2)	C5—H5	0.9500
C3—C4	1.459 (2)	C8—H8	0.9500
C3—C15	1.393 (3)	C9—H9	0.9500
C4—C5	1.319 (3)	C10—H10	0.9500
C5—C6	1.457 (2)	C11—H11	0.9500
C6—C7	1.401 (2)	C12—H12	0.9500
C6—C11	1.389 (3)	C13—H13	0.9500
C7—C8	1.386 (2)	C14—H14	0.9500
C8—C9	1.384 (3)	C15—H15	0.9500
C9—C10	1.373 (3)	C16—H16A	0.9900
C10—C11	1.372 (3)	C16—H16B	0.9900
C12—C13	1.382 (3)	C18—H18	0.9500
C13—C14	1.374 (4)		
			
C2—N1—C7	114.53 (14)	C3—C4—H4	117.00
C2—N1—C16	117.15 (13)	C5—C4—H4	117.00
C7—N1—C16	116.10 (13)	C4—C5—H5	117.00
N1—C2—C3	117.96 (14)	C6—C5—H5	116.00
N1—C2—C12	122.26 (15)	C7—C8—H8	120.00
C3—C2—C12	119.77 (16)	C9—C8—H8	120.00
C2—C3—C4	123.10 (16)	C8—C9—H9	120.00
C2—C3—C15	118.11 (17)	C10—C9—H9	120.00
C4—C3—C15	118.76 (17)	C9—C10—H10	120.00
C3—C4—C5	126.88 (16)	C11—C10—H10	120.00
C4—C5—C6	127.00 (15)	C6—C11—H11	119.00
C5—C6—C7	122.38 (16)	C10—C11—H11	119.00
C5—C6—C11	119.39 (16)	C2—C12—H12	120.00
C7—C6—C11	118.21 (15)	C13—C12—H12	120.00
N1—C7—C6	118.38 (14)	C12—C13—H13	120.00
N1—C7—C8	122.01 (14)	C14—C13—H13	120.00
C6—C7—C8	119.58 (15)	C13—C14—H14	120.00
C7—C8—C9	120.57 (16)	C15—C14—H14	120.00
C8—C9—C10	120.19 (17)	C3—C15—H15	119.00
C9—C10—C11	119.39 (18)	C14—C15—H15	119.00
C6—C11—C10	121.97 (18)	N1—C16—H16A	109.00
C2—C12—C13	120.44 (18)	N1—C16—H16B	109.00
C12—C13—C14	120.3 (2)	C17—C16—H16A	109.00
C13—C14—C15	119.5 (2)	C17—C16—H16B	109.00
C3—C15—C14	121.9 (2)	H16A—C16—H16B	108.00
N1—C16—C17	110.96 (14)	C17—C18—H18	180.00
C16—C17—C18	179.8 (2)		
			
C7—N1—C2—C3	69.0 (2)	C4—C3—C15—C14	−178.4 (2)
C7—N1—C2—C12	−112.4 (2)	C3—C4—C5—C6	−0.8 (4)
C16—N1—C2—C3	−149.95 (18)	C4—C5—C6—C7	32.0 (3)
C16—N1—C2—C12	28.6 (3)	C4—C5—C6—C11	−146.4 (2)
C2—N1—C7—C6	−72.36 (18)	C5—C6—C7—N1	7.6 (3)
C2—N1—C7—C8	110.12 (17)	C5—C6—C7—C8	−174.84 (18)
C16—N1—C7—C6	146.21 (16)	C11—C6—C7—N1	−174.02 (16)
C16—N1—C7—C8	−31.3 (2)	C11—C6—C7—C8	3.6 (3)
C2—N1—C16—C17	58.9 (2)	C5—C6—C11—C10	175.77 (19)
C7—N1—C16—C17	−160.75 (14)	C7—C6—C11—C10	−2.7 (3)
N1—C2—C3—C4	−2.9 (3)	N1—C7—C8—C9	175.51 (16)
N1—C2—C3—C15	178.7 (2)	C6—C7—C8—C9	−2.0 (3)
C12—C2—C3—C4	178.5 (2)	C7—C8—C9—C10	−0.6 (3)
C12—C2—C3—C15	0.1 (3)	C8—C9—C10—C11	1.6 (3)
N1—C2—C12—C13	−178.59 (19)	C9—C10—C11—C6	0.1 (3)
C3—C2—C12—C13	0.0 (3)	C2—C12—C13—C14	−0.3 (3)
C2—C3—C4—C5	−33.6 (4)	C12—C13—C14—C15	0.5 (4)
C15—C3—C4—C5	144.8 (2)	C13—C14—C15—C3	−0.4 (4)
C2—C3—C15—C14	0.1 (4)		

### Hydrogen-bond geometry (Å, º)

Cg1 and Cg2 are the centroids of the C2/C3/C12–C15 and C6-C11 rings, respectively.

**Table:**

*D*—H···*A*	*D*—H	H···*A*	*D*···*A*	*D*—H···*A*
C10—H10···*Cg*2^i^	0.95	2.75	3.659 (2)	160
C18—H18···*Cg*1^ii^	0.95	2.58	3.512 (2)	167

Symmetry codes: (i) −*x*, *y*, *z*−1/2; (ii) *x*, *y*, *z*+1.
